# IRF5 suppresses metastasis through the regulation of tumor-derived extracellular vesicles and pre-metastatic niche formation

**DOI:** 10.1038/s41598-024-66168-w

**Published:** 2024-07-05

**Authors:** Bailey K. Roberts, Dan Iris Li, Carter Somerville, Bharati Matta, Vaishali Jha, Adison Steinke, Zarina Brune, Lionel Blanc, Samuel Z. Soffer, Betsy J. Barnes

**Affiliations:** 1 Center for Autoimmune Musculoskeletal and Hematopoietic Diseases, The Feinstein Institutes for Medical Research, Manhasset, NY 11030 USA; 2 Elmezzi Graduate School of Molecular Medicine, The Feinstein Institutes for Medical Research, Manhasset, NY 11030 USA; 3 University of Notre Dame, Notre Dame, IN 46556 USA; 4 Department of Pediatric Surgery, Zucker School of Medicine at Hofstra-Northwell, Hempstead, NY 11549 USA; 5 Donald and Barbara Zucker School of Medicine at Hofstra/Northwell, Hempstead, NY 11549 USA; 6 Departments of Molecular Medicine and Pediatrics, Zucker School of Medicine at Hofstra-Northwell, Hempstead, NY 11549 USA

**Keywords:** Cancer, Immunology

## Abstract

Metastasis is driven by extensive cooperation between a tumor and its microenvironment, resulting in the adaptation of molecular mechanisms that evade the immune system and enable pre-metastatic niche (PMN) formation. Little is known of the tumor-intrinsic factors that regulate these mechanisms. Here we show that expression of the transcription factor interferon regulatory factor 5 (IRF5) in osteosarcoma (OS) and breast carcinoma (BC) clinically correlates with prolonged survival and decreased secretion of tumor-derived extracellular vesicles (t-dEVs). Conversely, loss of intra-tumoral IRF5 establishes a PMN that supports metastasis. Mechanistically, IRF5-positive tumor cells retain *IRF5* transcripts within t-dEVs that contribute to altered composition, secretion, and trafficking of t-dEVs to sites of metastasis. Upon whole-body pre-conditioning with t-dEVs from IRF5-high or -low OS and BC cells, we found increased lung metastatic colonization that replicated findings from orthotopically implanted cancer cells. Collectively, our findings uncover a new role for *IRF5* in cancer metastasis through its regulation of t-dEV programming of the PMN.

## Introduction

The transcription factor interferon regulatory factor 5 (IRF5) is a central mediator of the host immune response to pathogens and is implicated in the pathogenesis of many inflammatory and autoimmune diseases. Although most well-known for its role in innate and adaptive immunity, *IRF5* is also important in the cellular response to DNA damage and death receptor-induced apoptosis, and more recently as a tumor suppressor^1–6^. Moreover, direct relationships between loss of *IRF5* expression and accelerated tumor initiation and growth, increased metastatic burden, and worsened overall prognosis have been reported^1–4,7–10^. Mechanistic studies via overexpression and/or knockdown/knockout of *IRF5* in immortalized cancer cell lines reveal functional links between loss of *IRF5* and increased cell proliferation, migration, and invasion in vitro^1–3,7,10–12^, and in vivo^3,7^. Conversely, re-expression of *IRF5* in cancer cells that have otherwise ‘lost’ expression, results in regained cell growth control^1,3^. Utilizing data from The Cancer Genome Atlas of all patients with breast cancer (TCGA-BRCA), we identified a significant association between loss of *IRF5* expression and worsened prognosis for patients with breast cancer (BC)^2^. These findings were recently replicated in vivo in a mouse model of triple-negative BC revealing that re-expression of IRF5 in 4T1 cells that lack endogenous IRF5 led to an increased number of tumor infiltrating leukocytes with anti-tumorigenic function, reduced metastasis, and increased overall survival^3^.

BC is the most common cancer in women with increasing incidence and mortality^13^. In the U.S., women have a 1 in 8 lifetime risk of developing invasive ductal carcinoma (IDC) and greater than a third of patients with pre-cancerous ductal carcinoma in situ (DCIS) will develop into IDC^14^. Although the current 5-year relative survival of patients with localized BC is very good, approaching 100%, it drops to about 25% with metastatic disease^15,16^. Thus, understanding mechanisms of metastasis is vital to improving survival in these patients.

Given the clear association of loss of IRF5 expression with worsened prognosis and increased metastasis of BC, we aimed to explore this association in another tumor type with similar devastating effects from metastatic disease. Osteosarcoma (OS) is the most common malignant bone sarcoma in children and adolescents, which, like BC, often metastasizes to the lungs^17,18^. Even children who present with an isolated tumor are likely to have recurrence presenting with lung metastasis with or without chemotherapy^19^. Metastatic disease incurs significantly worse overall prognosis, dropping from 80% to about 20% and is associated with significant morbidity due to the need for pulmonary metastasectomie**s** and extensive systemic chemotherapy^20,21^. Due to significant disease heterogeneity, the burden of metastasis, metastatic recurrence, and resistance to current chemotherapeutic options, the need for improved understanding of the metastatic cascade cannot be understated for OS as well as BC.

Metastasis involves the dissemination of cancer cells from the primary tumor to distant organ sites and their adaptation to the new environment(s). This process is driven by extensive cooperation between a tumor and its microenvironment. Despite recent advances in understanding the molecular determinants that drive metastasis^16,22,23^, the identification of tumor-intrinsic factors and drivers of the crosstalk between tumor cells, the immune system, and the microenvironment remains poorly understood. Primary tumors can educate secondary sites through the release of soluble tumor-derived factors that enable crosstalk between the sites and the establishment of a pre-metastatic niche (PMN). The PMN is established through the intricate cross-talk between tumor-secreted factors, stromal components, bone marrow-derived mesenchymal stem cells, and suppressive immune cells^24^. Tumor-derived soluble factors, such as cytokines and chemokines, growth factors, and extracellular vesicles (EVs) signal to the microenvironment and allow the tumor to escape or be eliminated by the immune system, and/or induce mobilization and colonization to secondary sites. Some of these soluble tumor-derived factors will induce trafficking of tumor infiltrating lymphocytes. These tumor infiltrating lymphocytes can contribute either positively or negatively to tumor growth, metastasis, and patient outcomes, dependent on which population(s) of immune cells, immunosuppressive or immunogenic, infiltrate the tumor. Thus, identification of tumor-intrinsic factors that initiate and/or mediate such tumor-derived signaling is critical for the prevention and treatment of metastatic cancers.

EVs are considered one of the primary means of communication between distant sites in the body and are linked to metastatic spread^25,26^. They are small, 30–1000 nm in size, particles released from all cells, and are inclusive of exosomes, microvesicles, and nanoparticles^27^. EV cargo consists of proteins, lipids, DNA, mRNAs, and small miRNAs that have a unique chemical profile based on the cell(s) that released them^28,29^. The contents of EVs can be transferred to distant cells and change the genotypic and phenotypic function of the cell^30^. Thus, EVs are implicated in metastatic organotropism, immune suppression, angiogenesis, and immune escape that enables tumor growth and remodeling at secondary sites^31^. Given that EVs are currently being investigated in immune regulation, as diagnostic biomarkers of disease (in both BC and OS)^32–34^, and developed as EV-based targeted immunotherapies, we utilized a panel of OS and BC cell lines with varying metastatic potential and examined the contribution of IRF5 to EV-mediated PMN formation and metastasis.

## Results

### Loss of IRF5 is associated with worse overall survival, advanced cancer stage, and increased metastasis

Our lab and others have reported an association between loss of IRF5 expression and tumorigenesis^2–4,7^. Previously, we reported that normal breast tissue and tissue revealing atypical ductal hyperplasia (ADH) stained positive for IRF5 expression, whereas only ~ 38% of DCIS and ~ 10% of IDC retained IRF5 expression^2,4^. These findings were further corroborated by correlation analysis of *IRF5* transcript expression with disease-free survival in all BCs within The Cancer Genome Atlas (TCGA-BRCA) revealing that tumors in the lowest quartile of *IRF5* expression have a poor prognostic indicator with decreased survival^4^. Conversely, tumor tissue retaining the top quartile of *IRF5* expression positively correlated with increased overall and recurrence-free survival^2^.

**Figure 1:**
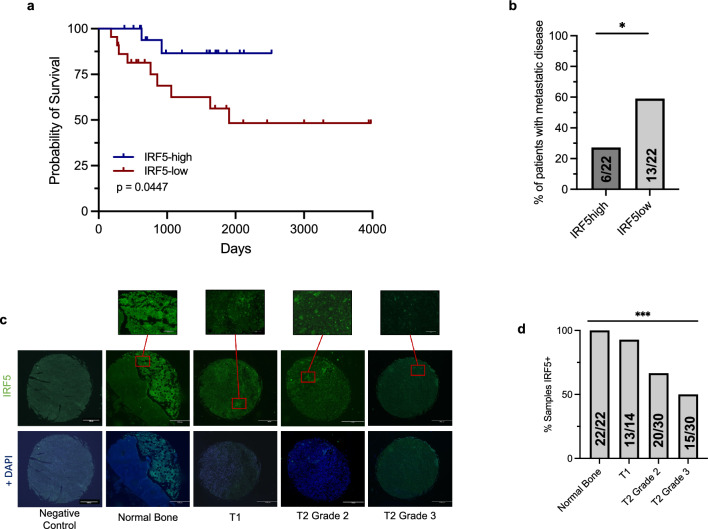
IRF5 is associated with better prognosis, decreased metastasis, and lower tumor grade in human OS. (**a**,**b**) Clinical and RNA-sequencing data from OS patients from the TARGET data matrix were stratified by IRF5 expression levels, metastases, and overall survival. Kaplan Meier curves were created for the bottom and top quartile of IRF5 expression via the UCSC Xena platform; n = 22 per group. (**b**) The top and bottom quartiles of *IRF5* expression were compared for the presence of patients with metastasis at diagnosis or development of recurrence at a metastatic site via a Chi-square test (*p* = 0.0334), n = 22 per group (**c**,**d**) Representative immunofluorescence staining of IRF5 in a panel of human OS tumor samples and normal bone controls (**c**). Quantification of the proportion of samples staining positive for IRF5 was compared using a Chi-Square test. Normal bone N = 22, T1 N = 14, T1 Grade 2 N = 30, T1 Grade 3 N = 30 (d). *p < 0.05, **p < 0.01.

Given these findings in BC, we next examined whether *IRF5* expression similarly correlated with overall survival in OS. The results published here are in whole or part based upon data generated by the Therapeutically Applicable Research to Generate Effective Treatments (www.cancer.gov/ccg/research/genome-sequencing/target) (TARGET) initiative, phs000468. The datasets analyzed during the current study are available in Genomic Data Commons repository for the Osteosarcoma TARGET Initiative (www.cancer.gov/ccg/research/genome-sequencing/target/studied-cancers/osteosarcoma). IRF5 quartiles and Kaplan Meier curves were created using the UCSC Xena platform^35^. The TARGET database had 88 patients with tumor RNA-sequencing data and matched clinical information. Loss of *IRF5* expression in OS correlated with worse overall survival in pediatric OS patients, and the top quartile of *IRF5* expression denoted those patients with better survival (*p* = 0.0447) (Fig. 1a). Presence of metastases at diagnosis or recurrence at metastatic sites was higher in patients with the lowest *IRF5* expression levels (*p* = 0.0331) (Fig. 1b). Last, by immunofluorescence staining, we confirmed the decrease in IRF5 expression in OS tumors of increased size and grade (*p* = 0.0033) (Fig. 1c,d).

### Murine intratumoral IRF5 is associated with decreased metastasis and predicts IRF5 transcript expression in tumor-derived EVs

**Figure 2:**
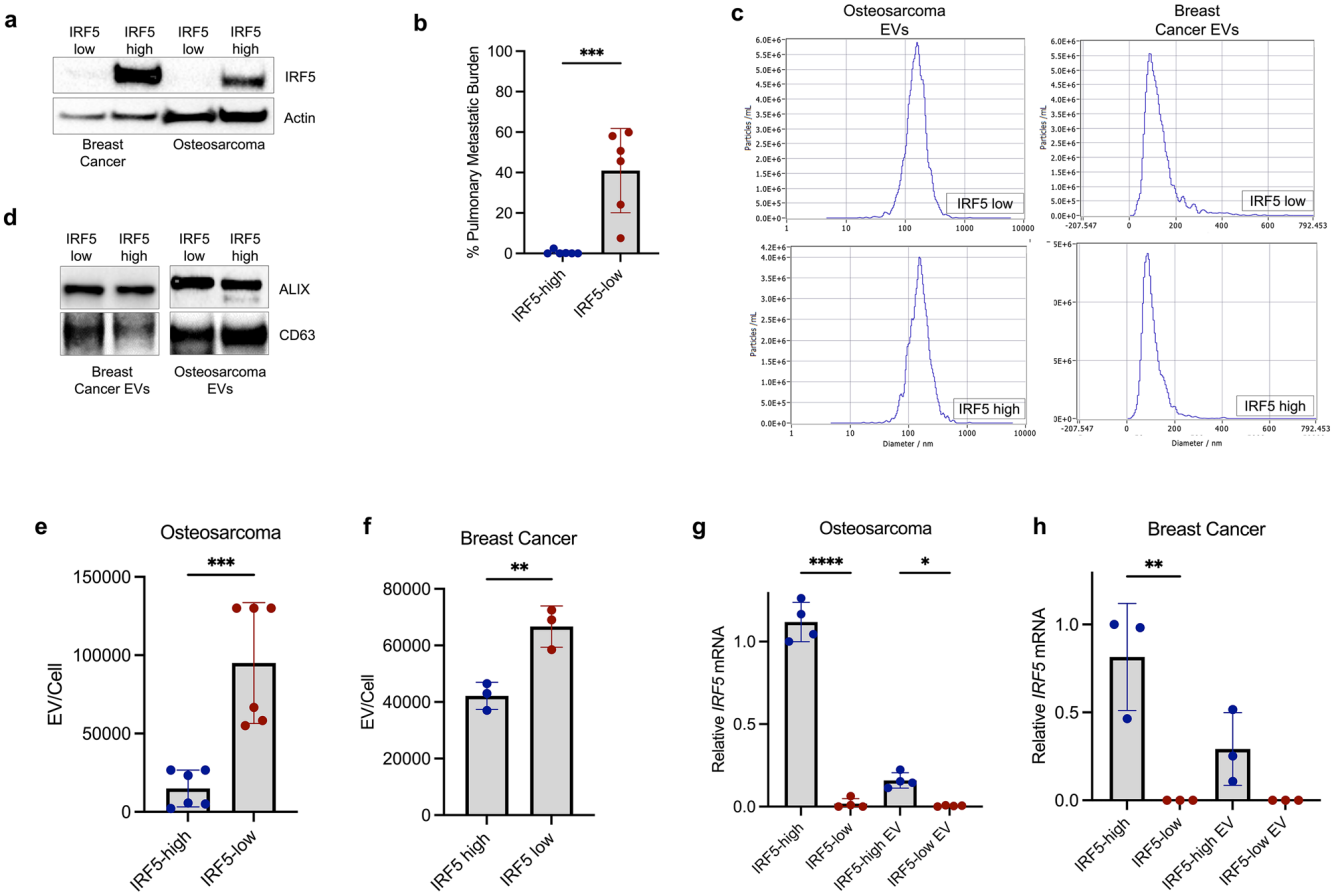
Murine intratumoral IRF5 is associated with decreased metastasis and predicts *IRF5* transcript expression in t-dEVs (**a**) Representative Western blot of IRF5 expression in 4T1 (IRF5 low) and 4T1-IRF5 (IRF5-high) expressing cells, and K12 and K7M2 osteosarcoma cell lines. (**b**) Pulmonary metastatic burden differences between K12 and K7M2 implanted tumors, N = 6 per group. (**c**) Representative size distribution of EVs isolated from the four cell lines. t-dEVs are between 0 and 200 nm. (**d**) Representative images of Western blots for ALIX and CD63 from t-dEVs. (**e**,**f**) Quantitative differences in EVs released per cell in K7M2 versus K12 and 4T1 IRF5-high and -low. (**g**,**h**) Relative *IRF5* transcript expression from qPCR analysis after normalization actin and to the cell with higher *IRF5* expression (K12, 4T1 IRF5 high). Western blots are representative of 3 independent replicates. Full Western Blot images for (**a**,**d**) are attached in Supplemental Fig. 1. *p < 0.05, **p < 0.01, ***p < 0.001, ****p < 0.0001.

Given these findings, two naturally occurring murine OS cell lines were evaluated for IRF5 expression. The K12 cell line is from a spontaneous OS in a Balb/c mouse with rare lung metastasis^36^, and K7M2 is a cell line of similar background created for increased metastatic potential and in vivo lung metastasis occurring ~ 100% of the time^36^. We previously reported that intratibial injections of K7M2 causes direct pulmonary seeding and is not a spontaneous model of metastasis^37^; yet, K12, a biologic variant of K7M2, forms tumors with similar potential but does not directly lead to pulmonary metastasis. This is likely an effect of tumor cell differences, the metastatic environment, and cross-talk from the primary tumor, and less from the implantation technique of the model^36,38^. Notably, K12 had high IRF5 expression, while K7M2 revealed little to no IRF5 expression by Western blot analysis, similar to the known difference in IRF5 expression from 4T1 IRF5-low and 4T1 IRF5-high cell lines^3^. (Fig. 2a). As expected, the cell line lacking IRF5 expression (IRF5-low) had the highest pulmonary metastatic burden after intratibial injection^36^. (Fig. 2b). These findings reflect the metastatic potential of IRF5-high and IRF5-low 4T1 triple-negative BC cell lines, where IRF5-low have high metastatic potential and IRF5-high show significant reductions in lung metastasis in Balb/c mice^3^.

Tumor-derived EVs (t-dEVs) are implicated in PMN formation^39,40^. We next isolated t-dEVs from the four cell lines, confirming size distribution by ZetaView nanoparticle tracking analysis, revealing similar size distribution with an average median size of 110 nm (Fig. 2c). We next confirmed expression of ALIX and CD63, proteins enriched in EVs and utilized as markers of EVs (Fig. 2d)^41–43^. Notably, we detected a significant decrease in EV secretion from cell lines that express IRF5; 4T1 IRF5-high and K12 (IRF5-high) revealed a 1.58-fold and 2.74-fold decrease, respectively, as compared to 4T1 IRF5-low and K7M2 (IRF5-low) (Fig. 2e,f). The concentration of EVs secreted from each cell line was determined and normalized to cell number. Given the difference of IRF5 protein in the cells, we next probed for IRF5 protein in the EVs, finding no evidence of IRF5 protein within the EVs from all four cell lines (data not shown). However, we noted *IRF5* transcripts were detected in t-dEVs from 4T1 IRF5-high and K12 (IRF5-high) cell lines (Fig. 2g,h). Together, these data suggest a potential new role for IRF5 in EV packaging and secretion.

### t-dEVs from highly metastatic IRF5-negative/low cell lines preferentially travel to sites of metastasis and increase metastatic burden

**Figure 3:**
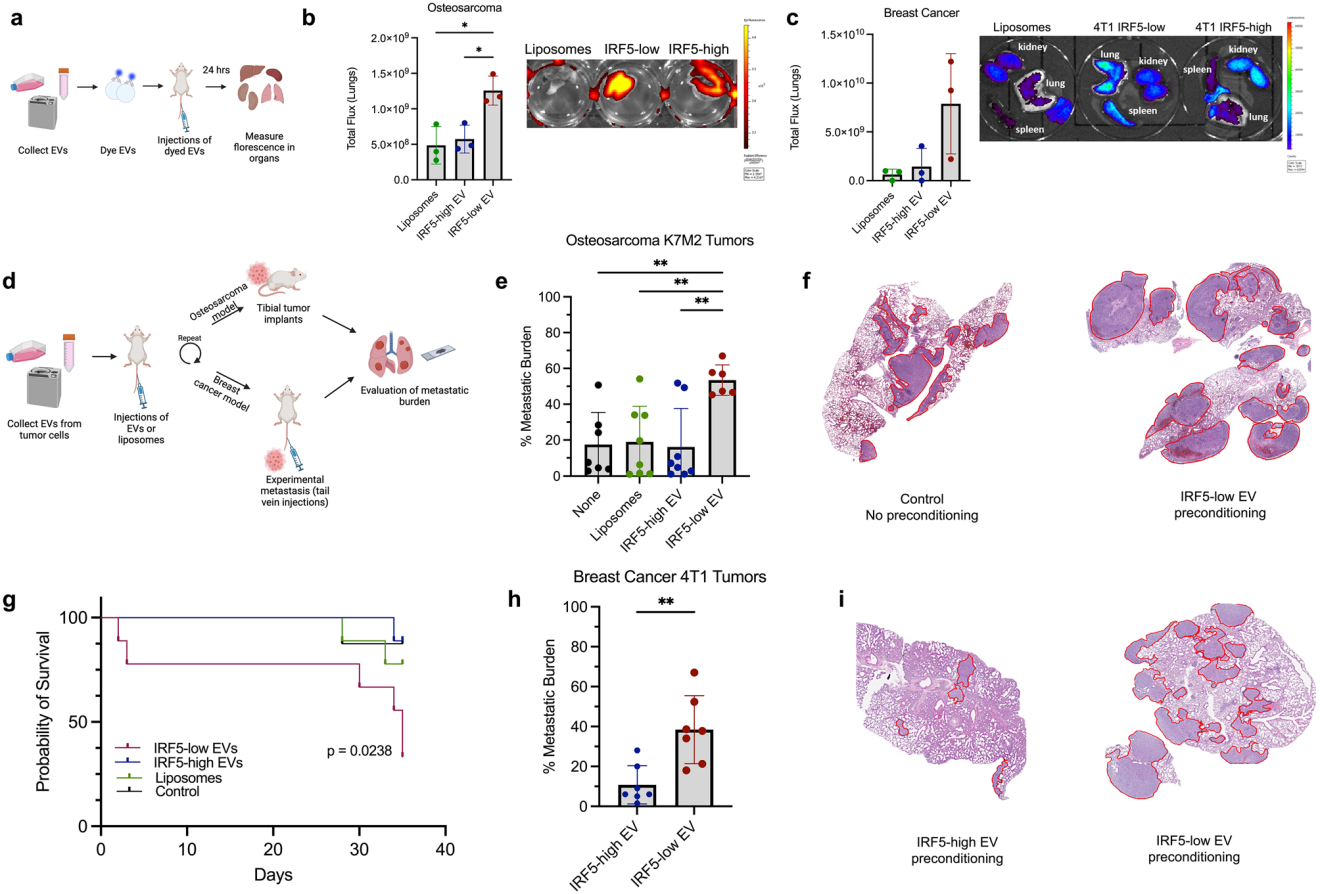
t-dEVs from OS and BC travel to the lungs in vivo, create a PMN, and increase metastatic burden. (**a**) Schema of in vivo EV tracking experiment. (**b**) Quantitation and representative image of total lung fluorescence after osteosarcoma EV injection. (**c**) Quantitation and representative image compared to other organs of total lung fluorescence after breast cancer EV injection. (**d**) Schema of EV pre-conditioning experiments. (**e**) Differences in metastatic burden of mice pre-conditioned with nothing, liposomes, K12 EVs, or K7M2 EVs prior to implantation of K7M2 tumors. (**f**) Representative H&E of metastatic burden in mice that were not pre-conditioned compared to K7M2 EV pre-conditioned mice. (**g**) Survival curves from the different EV pre-conditioning strategies over the time-period of our study, N = 8 per group. (**h**) Differences in metastatic burden of mice pre-conditioned with IRF5-high or IRF5-low EVs prior to IV injection of 4T1 cells. (**i**) Representative images of (**h**). (**a**,**d**) Created with Biorender. *p < 0.05, **p < 0.01, ***p < 0.001, ****p < 0.0001.

We next evaluated whether t-dEVs isolated from IRF5-high or -low tumor cells differentially traffic to organs. t-dEVs were isolated, fluorescently labeled, and equal numbers of labeled t-dEVs or liposomes intravenously (IV) injected into Balb/c mice. Organ deposition was determined 24 h later by bioluminescence imaging (Fig. 3a). We detected a significantly higher uptake of t-dEVs from 4T1 and K7M2 (IRF5-negative) cells in the lungs of Balb/c mice (Fig. 3b,c), while no significant difference in EV deposition at other organ sites was detected between groups (Supplemental Fig. 2a,b). Altogether, these data suggest that the observed differences in lung metastasis between IRF5-high and IRF5-low/negative tumor cells may be due to alterations in EV composition, function, and organotropism rather than differences in EV secretion since equal numbers of EVs were injected for trafficking experiments.

Given that t-dEVs isolated from IRF5-low/negative cells were found to preferentially travel to the lungs in vivo*,* we next assessed the contribution of t-dEVs to lung PMN formation by t-dEV “pre-conditioning.” As depicted in Fig. 3d, mice were pre-conditioned with equal numbers of t-dEVs or liposomes via tail vein injection prior to tumor implantation^44^. The incidence of lung metastasis was determined by H&E staining. For the OS model, EVs were injected 10 times over three weeks via tail vein and then K7M2 or K12 cells were intratibial-implanted for tumor formation^25,45^. Pre-conditioning of mice with EVs from K7M2 significantly increased the metastatic burden of K7M2 compared to baseline and K12 EVs (Fig. 3e,f). K7M2 EVs were also able to induce micrometastases of the poorly metastatic K12 (Supplemental Fig. 3). EVs from K12 did not lead to increased metastatic burden for K7M2 or K12 and thus were not able to create a PMN that supported lung metastasis (Fig. 3e). Further, pre-conditioning with K7M2 EVs led to significantly worse survival during the time-period of our study (Fig. 3g).

To more rigorously assess the contribution of IRF5 to t-dEV-induced metastasis, we performed similar experiments in the BC model. For the BC model, after t-dEV pre-conditioning with EVs isolated from 4T1 IRF5-low or 4T1 IRF5-high cells, mice were given IV injections of 4T1 cells for a purely metastatic model, and lungs were harvested 14 days later. As previously described, there is minimal metastasis noted at baseline for 4T1 IV injection of cells, with a noted increase in metastasis with 4T1 EV pre-conditioning^44^. We found that pre-conditioning with EVs derived from IRF5-high 4T1 cells resulted in significantly decreased lung metastatic burden compared to the increased metastasis of pre-conditioned 4T1 IRF5-low EVs, potentially exposing a protective role of IRF5 (Fig. 3h,i). These data replicate previously published work showing that 4T1-derived EVs support metastasis by conditioning metastatic organs with favorable PMN microenvironments^44^. Conversely, we found that EVs derived from 4T1-IRF5-high cells had significantly less lung metastasis and thus a PMN that is unable to support lung metastasis.

### IRF5 t-dEV immune cell reprogramming within the PMN

Emerging evidence indicates a role for t-dEVs in promoting metastasis through regulation or reprogramming of the tumor immune microenvironment^31^. Based on data in Fig. 3 showing that t-dEVs from IRF5-low OS and BC cell lines are capable of mediating a PMN that enables metastasis, we sought to characterize alterations in the lung stroma and immune microenvironment that may facilitate metastatic growth. To assess these early changes in the lung microenvironment, we performed multicolor flow cytometry at different stages of tumor growth—Days 1, 3, and 7 in mice implanted either with K7M2 or K12 cells. These days were chosen based on knowledge that some tumor cells do seed the lungs at the time of implantation and gross metastasis occurs by day 14. Conversely, the 4T1 mammary fat pad injection model does not cause direct pulmonary seeding and significant pulmonary metastasis does not occur until day 21 (Supplemental Fig. 4); thus, we chose days 3, 7, and 14 to represent the PMN in the 4T1 BC model. Given that previous studies in OS and BC have found alterations in myeloid cells within the tumor microenvironment that contribute to metastasis^46–52^, we sought to focus on these cell subsets in the lung microenvironment. In particular, myeloid derived suppressor cells (MDSCs) have been linked to metastatic progression through interactions with tumor cells at the metastatic site and suppression of T cells^50^. Further, t-dEVs have specifically been shown to target MDSCs^51,53^.

**Figure 4:**
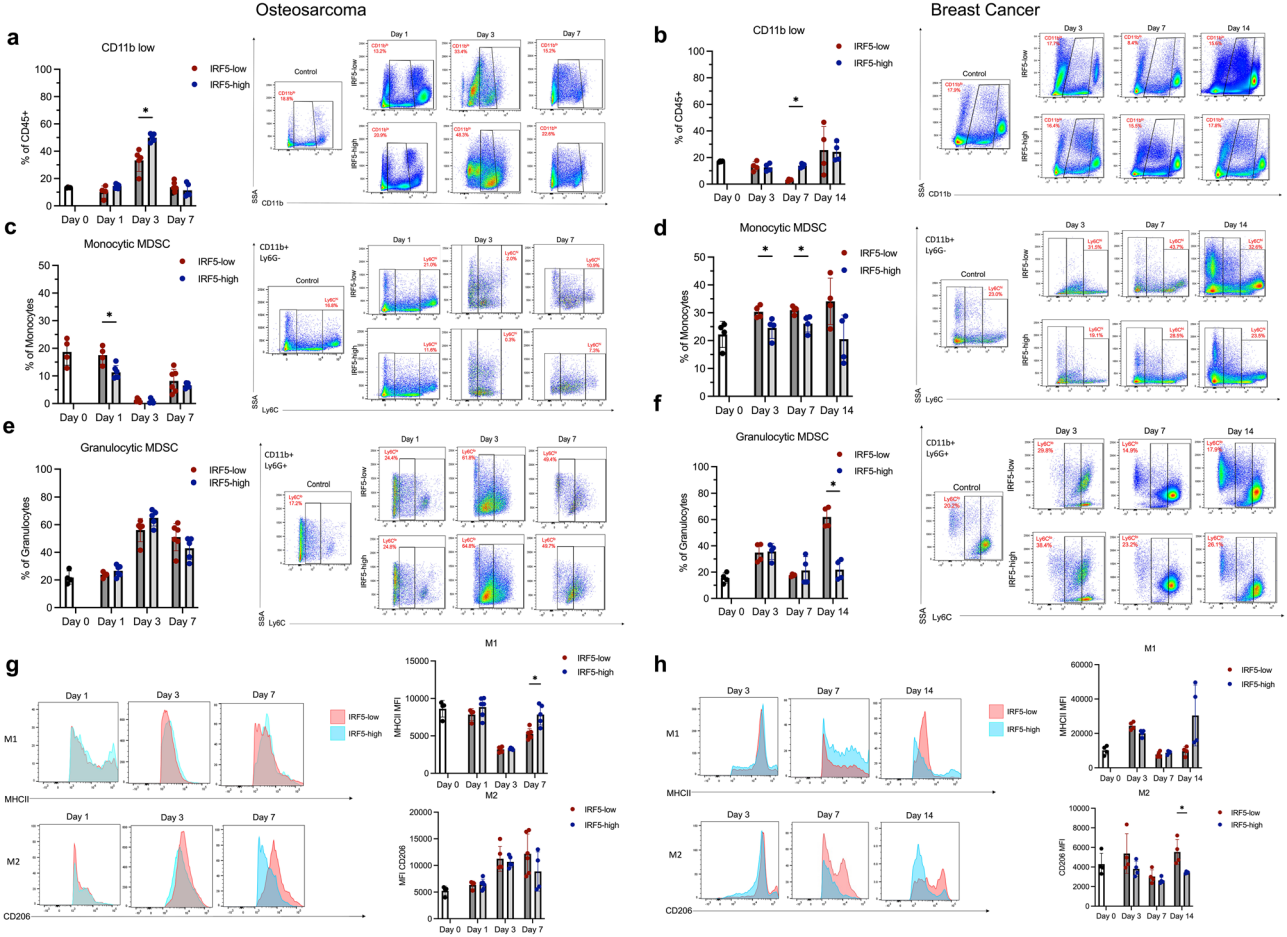
Immune cell reprogramming in the PMN is regulated by IRF5 t-dEVs. (**a**,**b**) Quantitation and representative flow cytometry gating of the CD11b^lo^ population during early stages of PMN formation from mice implanted with OS tumors (**a**) or BC implanted tumors (**b**). (**c**,**d**) Same as (**a**,**b**) except for CD11b^+^Ly6G^−^Ly6C^hi^, or monocytic MDSCs during early stages of PMN formation from OS implanted tumors (**c**) or BC implanted tumors (**d**). (**e**,**f**) Quantitation and representative flow cytometry of CD11b^+^Ly6G^+^Ly6C^lo^, or granulocytic MDSCs, during early stages of PMN formation from OS (**e**) and BC (**f**). (**g**,**h**) Representative flow histograms and quantitation of MFI for MHCII and CD206 during early stages of PMN formation in OS (**g**) and BC (**h**). *p < 0.05, **p < 0.01, ***p < 0.001, ****p < 0.0001.

No major difference in the infiltration of CD45^+^ leukocytes in the lungs of mice bearing either IRF5-high or IRF5-low tumors (BC or OS) was detected at any of the time points analyzed (Supplemental Fig. 5a). Focusing on CD11b^+^ myeloid cells revealed minor differences at the later stages of tumor growth in the BC model, but no other major differences between IRF5-high and IRF5-low at any timepoint (Supplemental Fig. 5b). While this trend was not seen at the earliest time points, we did notice expansion of a CD11b^lo^ population at Days 3 and 7 in mice carrying IRF5-positive OS or BC tumors that likely represent transitional myeloid cells (Fig. 4a,b). Examination of other cell types including neutrophils, total macrophages, and dendritic cells, as well as inflammatory subtypes of these cells, revealed no significant differences in early PMN stages (data not shown).

**Figure 5:**
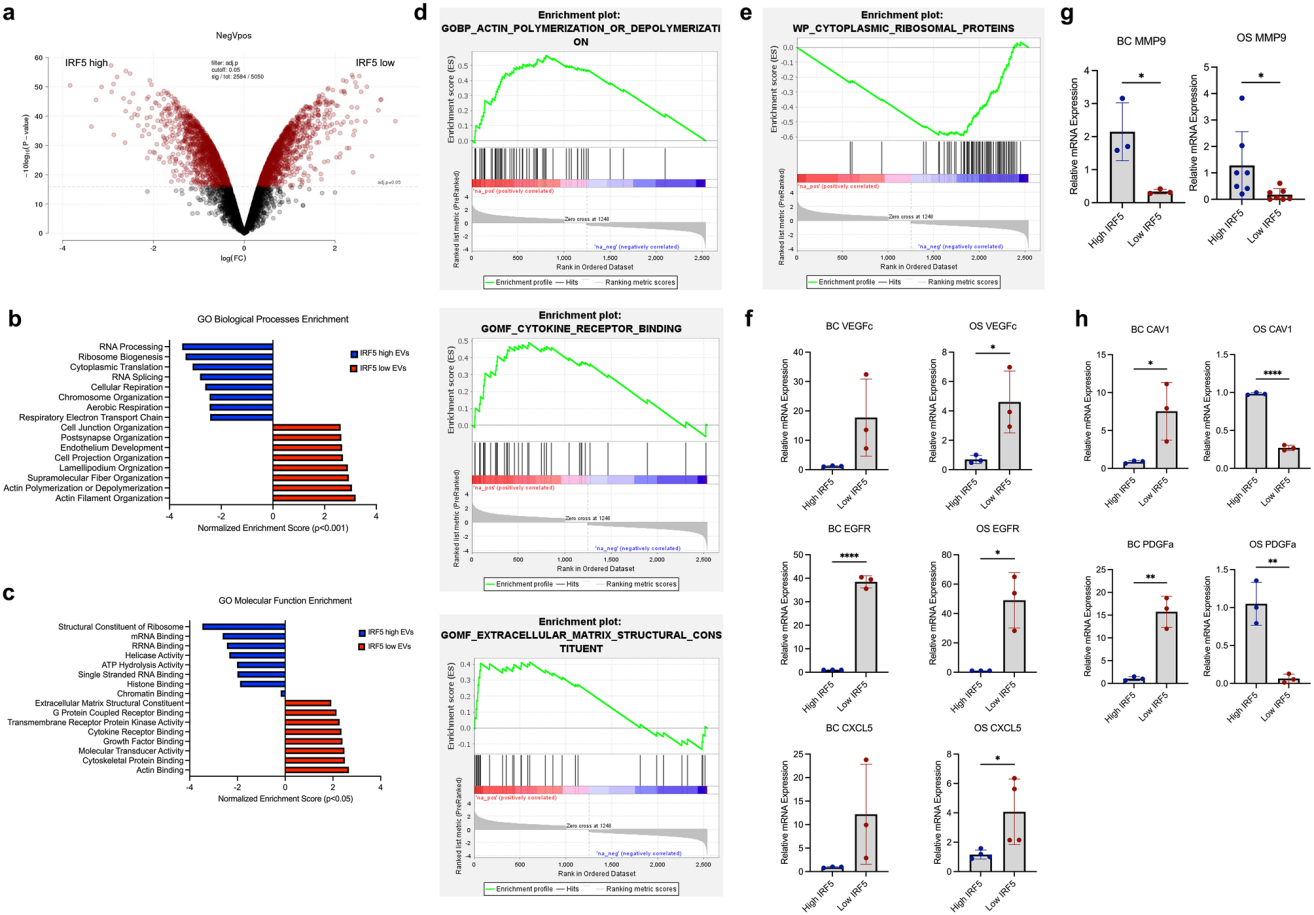
EV packaged protein differs in IRF5-high and IRF5-low t-dEVs. (**a**) Differential expression of 2584 proteins expressed in IRF5-high and IRF5-low t-dEVs from 4T1 cells; red indicates differential expression with *p* > 0.05 of N = 3 individual replicates. (**b**) Representation of normalized enrichment scores of the GO Biological Process pathways differentially enriched in IRF5-high and IRF5-low t-dEVs with *p* < 0.001. (**c**) Normalized enrichment scores of the GO Molecular Function pathways differentially enriched in IRF5-high and IRF5-low EVs with *p* < 0.05. (**d**) Representative plots of Actin polymerization, Extracellular Matrix Structure, and Cytokine Receptor Binding showing enrichment in IRF5-low t-dEVs. (**e**) Representative plot of cytoplasmic ribosomal proteins showing enrichment in IRF5-high t-dEVs. (**f**) Relative mRNA expression of genes enriched in IRF5-low EVs for both BC and OS, VEGFc, EGFR, and CXCL5. (**g**) Relative mRNA expression of MMP9, enriched in IRF5-high EVs. (**h**) Relative mRNA expression of genes enriched in IRF5-low EVs from BC cells, but not enriched in OS t-dEVs, CAV1, PDGFa. *p < 0.05, **p < 0.01, ***p < 0.001, ****p < 0.0001.

We examined infiltrating MDSCs that exist as two primary subsets: a monocytic subset defined as CD11b^+^Ly6G^−^Ly6C^hi^ and a granulocytic subset defined as CD11b^+^Ly6G^+^Ly6C^lo^^50^. The monocytic MDSC subpopulation was higher in the IRF5 low tumor bearing mice at early stages of tumor growth in both the OS and BC models (Fig. 4c,d), indicating a subpopulation of MDSC that may be implicated in metastatic growth from IRF5 loss. In the granulocytic subset, there were no major differences, although there was a trend toward an increase in the IRF5 low tumor bearing mice at later stages of PMN development (Fig. 4e,f).

Since IRF5 is a key mediator of macrophage polarization whereby IRF5 drives M1-like polarization, immunogenic macrophages, and loss of IRF5 drives M2-like, pro-growth, and pro-tumor macrophages, we examined macrophage polarization in the lung^54^. Although no significant differences were observed in the percentage of cells polarized towards M1- or M2-like between cell lines, there was a trend towards increased MFI of MHCII^+^ from IRF5-high tumors, representing an increase in M1 markers, and increased MFI of CD206^+^ from IRF5-low tumors, representing an increase in M2 markers (Fig. 4g,h). Altogether, these data show a contribution of myeloid derived suppressor cells and macrophage polarization in the differential establishment of a PMN from IRF5-high and IRF5-low tumors.

### IRF5 in tumor cells determines differential protein packaging in t-dEVs

Since we noted a divergence in the ability of the t-dEVs to establish a pre-metastatic niche, we sought to understand the differences in packaging of t-dEV cargo that may account for this variability. In order to elucidate the impact of IRF5 on t-dEV cargo, we focused our attention on the 4T1 BC model as it represents isogenic cell lines that only differ in their IRF5 expression. Thus, differences in t-dEV cargo may be directly related to differences in IRF5 expression, as compared to K7M2 and K12 cell lines that may have additional cellular and molecular differences^36^.

By proteomics analysis of t-dEVs isolated from 4T1 IRF5-high and -low cell lines, we identified 2,584 differentially expressed proteins in IRF5-low compared to IRF5-high EVs with differential expression of *p* < 0.05 (Fig. 5a). Of particular interest, we identified several proteins that were significantly enriched in IRF5-negative EVs that have been associated with more aggressive cancers and poorer prognoses. Following enrichment analysis, proteins that showed significant differential expression between the IRF5-high versus IRF5-low EVs (*p* < 0.05) were then further analyzed using GSEA Pre-ranked Gene Set Enrichment Analysis using the C2 curated gene sets. We identified significant differential expression of proteins in pathways constituting a variety of biological processes (Fig. 5b) and molecular functions (Fig. 5c) that support our prior findings that 4T1 IRF5-low BC is highly metastatic and t-dEVs from this cell line establish a PMN that increases metastasis. This is due in part to the finding of gene set enrichment in pathways involved in metastasis, PMN formation, immune cell trafficking and reprogramming, and actin cytoskeleton remodeling (Fig. 5d). Further, EVs from IRF5-high BC revealed an enrichment in proteins involved in ribosomal biogenesis and RNA processing (Fig. 5e). Proteins showing significant alterations with high enrichment scores and presence in multiple pathways were selected in the 4T1 IRF5-high and IRF5-low EVs. Proteins of interest included caveolin-1 (CAV-1) which showed enrichment in EVs from IRF5-low BC with a score of 3.328, *p* = 0.003. Platelet derived growth factor-A (PDGFa) was enriched in IRF5-low EVs with an enrichment score of 2.486, *p* = 0.003. Vascular endothelial growth factor C (VEGFc) was enriched in IRF5-low EVs with an enrichment score of 2.098, *p* = 0.010. Epidermal growth factor receptor (EGFR) was enriched in IRF5-low EVs with a score of 1.885, *p* = 0.003. CXCL5 was enriched in IRF5-low EVs with a score of 1.066, *p* = 0.010. Last, matrix metalloproteinase-9 (MMP9) was enriched in IRF5-high EVs with a score of − 2.332, *p* = 0.003. Given the low concentration of proteins that are packaged into EVs and the relatively greater amount of mRNA, these six proteins were assessed for their transcript expression in the BC EVs. We found conservation of the differences in protein and transcript between IRF5-high and IRF5*-*low BC EVs (Fig. 5f–h). Transcripts of these six proteins were also determined in OS EVs to assess the influence of IRF5 function in EV packaging on these proteins. We detected enrichment of *VEGFc*, *EGFR*, and *CXCL5* in the EVs from IRF5-low EVs cells for both BC and OS cell lines (Fig. 5f). Conversely, EVs from IRF5-high cells were enriched with *MMP-9* from both BC and OS cell lines (Fig. 5g). *PDGFa* and *CAV1*, however, were found to have an opposite enrichment in OS EVs compared to BC EVs, with enrichment in EVs from IRF5-low BC cells but enrichment in EVs from IRF5-high OS cells (Fig. 5h) Altogether, data confirm that t-dEVs from IRF5-low cancers are capable of altering the PMN specifically through alterations in myeloid immune cells, but the ability to create a PMN is not limited to immune cell trafficking and involves some alterations in extracellular matrix remodeling, migration, angiogenesis, and cell growth.

## Discussion

EVs have been implicated in promoting cancer progression and contributing to the metastatic cascade via direct interaction with immune cells and shifting of the immune microenvironment towards an anti-inflammatory phenotype in the PMN^31,55,56^. As EV cargo is unique and specific to the cell it is secreted from, t-dEVs can be important markers for prognosis and surveillance and may lend to novel treatment strategies. Further, the ubiquitous nature of t-dEVs enables them to travel through the bloodstream and re-locate to specific sites, making them a particularly interesting target of tumor metastasis. Primary tumors in both BC and OS are relatively well controlled with a combination of surgery, chemotherapy, and immunotherapies; the largest driver of death is metastasis. Given that metastatic disease determines long-term prognosis in patients with BC and OS, amongst other solid tumors, a thorough understanding of the factors implicated in metastasis will ultimately aid in early diagnosis and therapeutic inhibition.

Indeed, t-dEVs have already been implicated in PMN formation^25,26,31,39,40^. Wolf-Dennan et al.^47^ previously reported that t-dEVs from OS K7M3 cells, similar but with higher metastatic potential than K7M2, induce an M2-like phenotype in alveolar macrophages and increase pulmonary metastasis. Similarly, Macklin et al.^57^ established that in two different paired OS cell lines of varying metastatic potential, HOS and KHOS and LoMet-C6 and HiMet-C6, EVs from the higher metastatic cell line were able to induce metastatic phenotypes from the less metastatic cells. Conversely, Mazumdar et al.^58^ reported that EVs were not involved in the establishment of a PMN. However, these experiments were performed in SCID mice using the human-derived OS cell line 143-B, implying that a major mechanism by which t-dEVs induce a supportive PMN is through alterations in the immune microenvironment as SCID mice would lack a significant immune response. Importantly, in this same model, t-dEVs reprogrammed fibroblasts to be more like cancer-associated fibroblasts—providing evidence that EV influence is not limited to immune escape^59^. Further affirmation of EV influence on OS metastasis through immune regulation can be seen from proteomic analysis of EVs from the serum of dogs with early and late stages of OS and lung metastasis revealing select packaging of genes related to immune evasion and complement regulation within EVs^60^.

Similarly, EVs isolated from BCs were shown to induce a PMN and increase lung metastasis^44,61^. Wen et al.^44^ reported increased metastasis when pre-conditioning mice with t-dEVs isolated from EO771 and 4T1 cell lines before tumor injection. Additionally, when comparing 4T1 IRF5-low BC EVs to EVs derived from an unrelated non-metastatic BC cell line 67NR, they detected increased exosome secretion, lung organotropism, and equivalent uptake by immune cells^44^, which is nearly identical to our findings in 4T1 IRF5-low and 4T1-IRF5 high expressing cells. Notably, though, we observed decreased metastasis from 4T1 IRF5-low cells after pre-conditioning with 4T1 IRF5-high t-dEVs. The field of EV research in cancer has expanded over the past 5–10 years and many labs are interested in studying how EVs prime a PMN to enhance or inhibit metastasis. Most studies support a role for t-dEVs from highly metastatic tumors priming a PMN for metastasis^44,47,51,61^; however, the factors involved in the inhibition of metastasis by t-dEVs are less known. In a mouse model of metastatic melanoma, Plebanek et al.^51^ found that t-dEVs from a non-metastatic melanoma cell line resulted in EVs that blocked metastasis to the lung that is reminiscent of findings in our OS and BC models where IRF5 expression is retained.

The role of IRF5 has been increasingly explored in tumor development and metastasis due to its well-established role as a tumor suppressor in many cancers^1–4,6,7^. Notably, this is the first report of IRF5 as a prognostic indicator in OS. While IRF5 has distinct cell type-specific roles, its effect on the behavior of various cancers differs, including in some instances, malignancies in which it has been reported as an oncogene^62–68^. Thus, questions remain regarding the function of IRF5 in the primary tumor microenvironment, the PMN, and the metastatic site. Although IRF5 has been implicated in tumor immune cell trafficking, along with having direct intratumoral effects on cell migration and invasion^7^, the idea that IRF5 may also function as a mediator of EV packaging and secretion has important implications for metastasis.

MDSCs are intrinsically involved in the negative regulation of the immune response and thus play major roles in tumorigenesis. MDSCs are phenotypically heterogenous but are all immature myeloid cells able to suppress adaptive immune responses as well as non-immune related functions including tumor angiogenesis, invasion, and metastasis^69^. Specifically, the expansion of these early populations of myeloid cells, including a granulocyte subtype (CD11b^+^Ly6G^+^Ly6C^lo^) and a monocytic subtype (CD11b^+^Ly6G^-^Ly6C^hi^) that migrate to the tumor site, lead to the upregulation of immunosuppressive genes ARG1 and iNOS, suppress T cell activity, and ultimately differentiate into tumor associated macrophages^50^. We noted that EVs from IRF5-low tumors had increased proportions of MDSCs, particularly in early PMN formation. Wen et al.^44^ noted similar MDSC expansion with 4T1 EVs compared to a distinct non-metastatic BC. Our work corroborates these findings, yet adds a slightly different perspective on the link between IRF5 expression, metastasis, and t-dEV-mediated PMN formation. Conversely, Plebanek et al.^51^ noted that the protective effects of less metastatic cell lines were through activation of patrolling undifferentiated monocytes by EVs.

Our data thus far suggests that one of the mechanisms by which EVs expressing *IRF5* (mRNA) exert their effects is through the alteration of these MDSCs and other differentiated myeloid cells. IRF5 promotes differentiation from monocytes to macrophages, decreasing the proportion of MDSCs^70^. Within these differentiated macrophages, IRF5 is known to alter the polarization of macrophages from M2 towards M1; in that IRF5 expression directly drives M1 polarization, while lack or downregulation of IRF5 induces M2 polarization^71,72^. Generally, M1 polarized macrophages are useful in immune activating functions including recruitment of cytotoxic T cells and other antigen presenting cells, and lead to apoptosis and cancer cell autophagy. Conversely, M2 polarization is associated with cancer promoting activity such as immune escape, proliferation, angiogenesis, tissue growth, and metastatic invasion. The link between t-dEVs and tumor associated macrophages has been previously demonstrated in both OS and BC^47,73^. The presence of IRF5, though, pulls myeloid cells and macrophages away from a tumor promoting state, releasing inflammatory cytokines, and regulating T cell activation and tumor killing^54,74^. Further, IRF5 is critical in dendritic cell activation. IRF5 is already highly expressed in dendritic cells and is required for TNF release, which enhances tumor cell killing^75,76^. Thus, EVs carrying *IRF5* may activate the local microenvironment to be more “anti-tumor” whereas the lack of IRF5 creates a “pro-tumor” microenvironment with increased MDSCs, TAMs, and inhibition of activated T cells. Further evidence confirming the role of myeloid based immune modulation is the observed upregulation of *CXCL5* in both the BC and OS IRF5-low EV, a chemokine known to recruit MDSCs, and thus may be instrumental in understanding the mechanism of loss of IRF5 and MDSC recruitment (Fig. 5).

Interestingly, MMP9, a molecule known for its role in the degradation of the extracellular matrix, has both pro-inflammatory and anti-inflammatory properties. Thus, it has been a target of interest to inhibit tumor progression. The role of MMP9 in tumor progression is controversial and differs by tumor and cell type. Further, clinical trials of MMP9 inhibitors have failed to show improvement in cancer progression^77^. However, in BC, MMP9 has previously been shown to induce inflammation that leads to tumor regression, specifically through increased neutrophil infiltration and induction of pro-inflammatory macrophages that drive anti-tumorigenic properties^78^. Upregulation of MMP9 by IRF5-high EVs may contribute to the pro-inflammatory environment that results in decreased metastasis.

Capitalizing on the ability of IRF5 to promote the polarization of macrophages and immune activation, the packaging of IRF5 into small nanoparticles for therapy has been trialed. A recent study by Zhang et al. reported on the loading of nanoparticles with *IRF5* mRNA and IKK-beta, a kinase known to activate IRF5^79^, created to target M2 macrophages^80^. These nanoparticles when delivered in vivo, successfully reduced metastatic burden in a murine model of peritoneal ovarian cancer, metastatic melanoma, and glioma. Subsequent studies by Gao et al.^81^ loaded *IRF5* into a nanoparticle with siRNA for CCL5, designed to target M2 macrophages. The nanoparticles recruited cytotoxic T cells, increased tumor cell killing, and reduced tumor burden in a murine model of pancreatic cancer. Our model of t-dEVs from IRF5-high and IRF5-low tumor cell lines provides additional pre-clinical evidence for altering the landscape of a tumor microenvironment and its PMN by packaging *IRF5* into EV-like nanoparticles for therapeutic delivery. Further, findings from the current study support the concept that *IRF5* mRNA within a t-dEV may be a biomarker of less metastatic disease and define a pathway to inhibit metastasis.

Other differentially regulated pathways related to metastasis and the establishment of a PMN include the actin cytoskeleton regulation and specifically regulation of proteins contributing to cell growth, tissue remodeling, and angiogenesis. The proteins differentially packaged into 4T1 IRF5-low and 4T1 IRF5-high EVs can mechanistically explain the observed disparate effects on the PMN. Four distinct proteins known to be involved in metastasis, VEGFc, EGFR, CAV-1, and PDGFa were found to be upregulated in IRF5-low BC EVs, implicating their potential role in PMN establishment. Two of these, VEGF and EGFR, were also enriched in the IRF5-low OS EVs. Both are ubiquitously expressed oncogenes. VEGF secreted by cancer cells leads to proliferation of endothelial cells and neovascularization, enhancing growth of the tumor^82^. In particular, the subtype VEGFc has been implicated in promoting BC tumor growth and metastasis, and its expression is also associated with worsened tumor grade in OS^83,84^. EGFR is widely accepted as a driver of tumorigenesis through a variety of downstream signaling cascades^85^. Thus, this oncogenic protein is also a target for treatments including monoclonal antibodies and vaccines^86^. Conversely, CAV-1 and PDGFa were noted to be expressed in IRF5-high EVs in OS versus IRF5-low EVs in BC. CAV-1 is a protein intricately involved in cellular signaling, differentiation, proliferation, and cell death, and paradoxically has been reported to be downregulated in the early stages of tumorigenesis but then re-expressed in later stages supporting invasion and proliferation^87^. Specifically in BC, CAV-1 expression is associated with worse disease free survival and overall survival in human breast cancer patients, and CAV-1 in EVs promoted breast cancer invasiveness in a murine model with MDA-MD-231 cells^88,89^. Interestingly, in OS, CAV-1 has been implicated as a tumor suppressor in older age OS patients and is associated with improved overall survival in a study with 6 year follow up^90,91^. CAV-1 is highly expressed in osteoblasts, and loss of CAV-1 has been thought to initiate transformation into malignant cells. Given the dual nature of CAV-1 as a known oncogene and tumor suppressor, the influence of the cell type on its role in tumorigenesis may be more influential than the impact of IRF5 on its regulation. Another gene noted to be enriched in BC IRF5-low EVs that was inversely enriched in OS EVs was PDGFa. PDGFs are very well studied as they are intricately involved in cell growth and survival, as well as metastasis, invasion, and angiogenesis^92–94^. Specifically, PDGFa mediates myofibroblast and epithelial proliferation. In breast cancer, PDGFa overexpression has been linked to metastasis, tumor aggressiveness, and progression^95^. Interestingly, this same association is not known in osteosarcoma. The role of PDGF in tumor proliferation in osteosarcoma has not been elucidated. While still understood that it is associated with worse prognosis, the previous studies show significant heterogeneity in its expression, ranging from 4 to 90%, indicating that PDGF may not be the dominant pathway by which osteosarcoma cells have malignant potential^96^. Further research determining the interplay between IRF5 and these proteins in different tumor cells is necessary to elucidate the impact of IRF5 on malignant potential. Nevertheless, the differential expression of EGFR and VEGFc, in addition to MMP9 and CXCL5, differentially packaging into IRF5-low versus IRF5-high EVs across two cancer types provide insight into the mechanisms by which IRF5 contributes to protection from PMN formation.

### Limitations

While our findings show that EVs from IRF5-high and -low tumor cell lines differ in function and effect on the PMN, the differences in metastatic potential associated with these t-dEVs may not be entirely related to IRF5. EVs carry a myriad of cargo with discrete roles for cellular communication by a multitude of molecules, not just one^97,98^. Thus, the direct role of *IRF5* transfer from EVs to cells in the PMNs needs further study. We also noted a difference in the quantity of EVs released from our IRF5-high and -low cell lines, which also may impact varying effects at the PMN*.* We did attempt to control for this when studying functional EV differences as we injected equal numbers of EVs for each experiment. Further, much of our focus was on the specific immune programming effects of t-dEVs on the lungs due to the role of IRF5 as a transcription factor that mediates immune activation and recruitment of tumor infiltrating lymphocytes^7,99,100^. However, it is likely that a combination of downstream mechanisms, including angiogenesis promotion, stromal activation, and induction of more aggressive tumor characteristics in addition to PMN formation. For example, both OS and BC EVs have been linked to creation of cancer associated fibroblasts, leading to increased metastasis, which were not examined in our study^59,101^. Some of these differences were noted in the proteomics, linking possible mechanisms of PMN formation to molecules such as EGFR, PDGFa, or VEGFc. A more thorough understanding of the mechanisms by which IRF5 alters EV release from cancer cells and leads to a protective metastatic environment is also warranted. Further, the proteomics data revealed notable differences in the BC IRF5-high and IRF5-low EVs, which were validated at the transcript level. It is possible that protein differences in EVs do not match transcript differences as there is selective packaging in these small nanoparticles. Indeed, we detected IRF5 protein within the cells but only *IRF5* transcripts within EVs, supporting the idea of selective packaging. The differences in packaging may thus lie in size discrepancy as proteins are much larger than mRNAs, limiting the amount that may be loaded. Similarly, many researchers focus on miRNAs as they are numerously packaged into EVs due to their size, but EVs have a mix of short and long mRNAs, proteins and other cargo^102^. Regardless of whether mRNA or protein is selectively packaged into EVs, there may be no functional difference on recipient cells as mRNAs from EVs have been shown to be delivered and successfully translated in recipient cells, including tumor cells, immune cells, and other cells in the microenvironment^103^. Given that IRF5 is a widely expressed protein/mRNA within immune cells of the PMN, it is challenging to detect direct transfer of contents such as *IRF5* from t-dEVs to recipient cells. However, the fact that we were able to detect increased M1-like macrophages within the PMN of mice carrying IRF5-high tumors suggests that *IRF5* transcripts were transferred. Consequently, additional research on the role of IRF5 in differential loading of protein and nucleic acids into EVs should be explored. Further, examining these findings in other models of BC and OS would be important to corroborate findings. Nevertheless, functional t-dEV differences from IRF5-high and -low tumors were established in two distinctly different tumor types, providing the necessary support for focusing on IRF5-mediated immune protection and targeting immune escape at distant sites via loss of IRF5 expression/function.

### Conclusion

In conclusion, our findings show that IRF5 expression in tumor cells translates to the packaging of *IRF5* mRNA in EVs, causing downstream effects of reduced metastatic burden and immune programming towards a more protective or anti-tumorigenic immune microenvironment. This is seen both in an isogenic cell line in which the only difference is ectopic IRF5 expression (4T1 IRF5-low and IRF5-high) and in two naturally occurring high and low metastatic OS cell lines (K7M2 and K12). Elucidating the function(s) of *IRF5* within t-dEVs, and/or its contribution to the select packaging and secretion of t-dEVS, will provide critical insight into the clinical utility of *IRF5* as a new therapeutic strategy to potentiate immunotherapy and inhibit metastasis of BC and OS, amongst others.

## Methods

### Cell lines and tissue culture

The murine 4T1 mammary carcinoma cell line and the Phoenix viral packaging cell line were purchased from American Type Culture Collection (ATCC). The luciferase-expressing 4T1 (4T1-Luc3) cell line was provided by Dr. Cheryl Jorcyk (Boise State University). All cell lines were confirmed negative for pathogens by Radil^®^ testing (IDEXX BioAnalytics; Westbrook, ME, USA). The K7M2 murine OS were purchased from ATCC (CRL-2836). K12 murine OS cell line was donated from the lab of Dr. Kurt Weiss at the University of Pittsburgh Medical Center. Cells were cultured in Dulbecco’s Modified Eagle Medium (DMEM) (Sigma-Aldrich, D0822) supplemented with 10% heat-inactivated premium Fetal Bovine Serum (Corning, 35-016) and 1% penicillin/streptomycin (Hyclone Laboratories). Cells were split using 0.05% trypsin (Cytvia, SH30236) when they reached 80% to 90% confluence.

### Mice

This study was carried out in strict accordance with recommendations in the *Guide for the Care and Use of Laboratory Animals* of the NIH^104^. The protocol was approved by the Institutional Animal Care and Use Committee (IACUC) of the Feinstein Institutes for Medical Research and the USAMRDC Animal Care and Use Review Office (ACURO). Male and Female Balb/c mice were purchased from JAX Laboratories and used for experiments at 8 to 12 weeks of age. For IV injections, the animals were warmed for 5–10 min to dilate the veins using a water circulating pad placed under the cage. After anesthesia with isoflurane 2%, a 25 µL suspension of 1.0 × 10^10^ or 5 µg of EVs were injected into the lateral tail vein. For tumor implantations, K12 or K7M2 cells at a concentration of 3.0 × 10^5^ cells in 10 µL of PBS were injected into the tibia as previously described^36,46,105^. For the BC model, 2.5 × 10^5^ cells were injected IV for an experimental model of metastasis. Post-operative monitoring of mice, including collection of body weight and assessment of pain, occurred daily for 3 days after injection and twice weekly thereafter. Based on previous studies, at 5 weeks post-injection many of the OS bearing mice meet euthanasia criteria, with large tumors, weight loss, and general ill health, and therefore all studies were terminated no later than 5 weeks after implantation. Similarly, the metastatic BC model was terminated at 2 weeks after implantation.

### Extracellular vesicle isolation and quantification

EVs from 4T1-Luc and 4T1-IRF5 as well as K7M2 and K12 cells were purified by ultracentrifugation of cell culture supernatants after 48 h culture. Briefly, cells were plated in T-125 flasks at a concentration of 1–1.5 × 10^6^ cells and allowed to grow to around 60% confluence. The media was changed to DMEM containing 10% EV Free FBS and 1% penicillin/streptomycin and were incubated for 48 h. Media was collected from cells and serially centrifuged to remove cells, debris, and macrovesicles at 1000G for 10 min, 2000G for 20 min, and 10,000G for 30 min collecting the media and discarding the pellet. Finally, the EVs were collected via ultracentrifugation at 100,000G for 2 h. EVs were resuspended in DPBS and passed through a 0.22 µm sterile filter before being washed and centrifuged again at 100,000G for 70 min. Quantification and size distribution of isolated EVs were determined using the ZetaView Nanoparticle Tracking Analysis (Particle Metrix, Basic NTA-Nanoparticle Tracking Video Microscope PMX-120) equipped with a blue laser (405 nm). All samples were diluted in PBS to give 20–100 particles/frame, according to the manufacturer’s software manual. After quantification, EVs were resuspended in DPBS to the proper concentration for mouse injections or further experiments.

### Immunofluorescence

Normal bone tissue array and OS panel tissue array were purchased from www.tissuearray.com (BO244g, OS804d). Cores from patient samples are plated on one microscopy slide and fixed with paraffin with matching pathology diagnosis and TNM and clinical staging information. Antigen retrieval was performed by heating slides at 95 °C in citrate buffer (pH 6.0) for one hour before staining with mouse anti-IRF5 (ab181553) at 1:100 dilution in 4% BSA/PBST overnight, as previously described^2^. Slides were incubated with anti-rabbit-AF488 (Invitrogen A11034) at 1:1000 in 4% BSA/PBST for one hour. Slides were mounted with DAPI mounting buffer (Vector laboratories) and images were captured on an EVOS microscope with GFP light. Quantification was performed using ImageJ software (Version 1.52a).

### Western blot

Cells and EVs were lysed in RIPA buffer (Pierce) supplemented with protease inhibitor (complete mini protease inhibitor cocktail 1:100 dilution, Roche Diagnostics). Protein concentration was measured by DC assay. Equal amounts of protein were resolved by electrophoresis on NuPAGE 8% Bis–Tris gels (Invitrogen) and transferred to PVDF membranes (ThermoFisher Scientific). Membranes were blocked with 5% BSA/TBST before probed with primary antibodies diluted 1:2000 anti-ALIX (BioRad MCS2493), 1:1000 anti-CD63 (Abcam ab213092), 1:1000 IRF-5 (Abcam ab181553) or overnight followed by incubation with 1:10,000 HRP-linked anti-rabbit (Cell Signaling Technology CST7074) or HRP-linked anti-mouse (Cell Signaling Technology CST7076) for one hour. Images were read on Bio-Rad ChemiDoc Imager.

### Polymerase chain reaction

RNA was isolated from cells with RNEasy Mini Kit (Qiagen) following the manufacturer’s instructions. cDNA was obtained using GoScript Reverse Transcriptase kit (Promega). Semiquantitative RT-PCR was performed using a LightCycler 480 system with the LightCycler 480 SYBR Green I master mix according to standard procedures (40 amplification cycles) (Roche). Primers for IRF5 (Forward sequence 5′-CCTACAGAACCACTCTTGCCTG-3′ and Reverse sequence 5′-CCTTGTGGGTTGCTGATGGTGA-3′), CAV1 (Forward sequence 5′-ACTTCCCAGCTCACATTACAG-3′ and Revere sequence 5′-AGTCAAGCAGGGTTCCAATAC-3′), EGFR (Forward sequence 5′-GCCATCTGGGTACGTTCAAT-3′ and Revere sequence 5′-GGAAGAAACTGGAAGGTGAGAG-3′), PDGF-a (Forward sequence 5′-TCCAGCGACTCTTGGAGATA-3′ and Revere sequence 5′-TCTCGGGCACATGGTTAATG-3′), VEGFc (Forward sequence 5′-CCACGTGAGGTGTGTATAGATG-3′ and Revere sequence 5′-CGGACACACATGGAGGTTTA-3′), CXCL5 (Forward sequence 5′-TGCCTGAAGGAAGAGAGAGA-3′ and Revere sequence 5′-TGGAGGAGGTGTGGAGATT-3′), and MMP-9 (Forward sequence 5′-GCTGACTACGATAAGGACGGCA-3′ and Revere sequence 5′-TAGTGGTGCAGGCAGAGTAGGA-3′) were used and housekeeping gene beta-actin (Forward sequence 5′-GGCTGTATTCCCCTCCATCG-3′ and Reverse sequence 5′-CCAGTTGGTAACAATGCCATGT-3′) for normalization control^106^. Experiments were performed in triplicate with n = 3 wells per experiment group.

### Murine fluorescent imaging

EVs were collected as previously described. EVs and liposomes were dyed with DiD (Invitrogen, V22887) at 5 µL/1 mL for 45 min at 4C. EVs were washed with PBS and ultracentrifuged at 100,000G for 1 h and resuspended to a concentration of 5.0 × 10^10^ EVs or liposomes/20 µL. 20µL of EVs or liposomes were injected via the tail vein of male and female Balb/c mice aged 8–12 weeks. After 24 h, mice were euthanized, and organs were harvested to image on IVIS Living Image (Caliper LifeSciences). Total flux was measured using IVIS Living Image Software.

### Lung colony formation assay

Clonogenic assays were performed as previously described^107^. Briefly, lungs were harvested from the mice and rinsed with PBS prior to mincing and treating with 2 mg/mL of collagenase type IV at 37C for one hour. Digested organs were passed through a 70 µm strainer, centrifuged, and resuspended in PBS. After two additional washes, whole organ suspension was then resuspended in RPMI supplemented with 10% fetal bovine serum and 60 µM 6-thioguanine (Fisher) and plated into 6-well plates. Plates were incubated at 37C with 5% CO2 for 10 days without media change. After incubation, the unattached cells were removed, and the plates were washed with PBS. Colonies were fixed with methanol for 1 h and then stained with 0.5% crystal violet for 2 h. The wells were rinsed and viewed under a microscope, counting the number of colonies formed.

### Assessment of lung tissue for pulmonary metastases

Mice were euthanized by CO2 asphyxiation, lungs were dissected out of the thorax and fixed with 10% neutral-buffered formalin and embedded in paraffin. Incidence of lung metastases was detected by histopathologic assessment. Paraffin embedded specimens were serially cut in longitudinal, 5 µm thick sections and stained by routine hematoxylin and eosin methods. To assess pulmonary metastases, each slide was examined microscopically under low power (0.63–20x) on an EVOS microscope with to visualize the lung section per microscopic field. Metastatic burden was calculated by measuring the average surface area of metastatic lesions in the lung divided by the total surface area of the lung per section using ImageJ software (Version 1.52a).

### Premetastatic niche formation and experimental metastasis studies

To initiate PMN formation, 8–12-week-old Balb/c mice were injected IV (tail vein) with 5 µg of purified EVs/injection, every 3 days for 30 days for the BC model, and 1 × 10^10^ EVs/injection a total of 10 times over 3 weeks for the OS model^25,44^. Control mice received an equivalent amount of liposomes. EVs were freshly isolated for each injection from either 4T1 and 4T1-IRF5 or K7M2 and K12 for injections. After pre-conditioning, mice were injected IV (tail vein) with 2.5 × 10^5^ 4T1 cells (experimental metastasis model) and metastatic burden in the lung was assessed 14 days later or intratibial tumor implantation into the left hind tibia with 3.0 × 10^5^ of K7M2 or K12 cells for the OS model and metastatic burden in the lung analyzed 5 weeks later^37,46^. Upon sacrifice, organs were harvested and either fixed for histopathological analysis or single-cell preparations generated for immune cell composition analysis by flow cytometry.

### Flow cytometry

Flow cytometry was carried out on single-cell suspensions of lung tissue. A standard protocol was used to enzymatically digest lung tissue and prepare single-cell suspensions for flow cytometry analysis of total lung tissue as previously described^108^. Flow cytometric acquisition was completed using LSR-Fortessa (BD Biosciences), and analysis was performed using FlowJo (Tree Star).

### Proteomics analysis

EVs were collected from 4T1 IRF5-low and 4T1 IRF5-high in three process replicates as previously described. Samples were sent to the Broad Institute for processing. Employed TMT6 labeling strategy and basic pH reverse phase fractionation for analysis of samples by LC–MS/MS. A one sample t-test was performed by taking the ratio of TMT channels corresponding to each IRF5−/IRF5 + sample preparation. Additionally, single sample gene set enrichment analysis was conducted using Log10 transformed *p*-values derived from the t-test.

### Statistical analysis

Quantitative data are presented as mean ± SEM. Frequency differences between groups were compared with Chi-Squared test. Non-parametric data were analyzed by two-tailed Mann–Whitney U tests. Parametric data were analyzed using t-tests and ANOVA with post hoc comparison (Tukey method). Survival curves were analyzed using the log-rank Mantel Cox test. Adjusted *p* < 0.05 was considered statistically significant.

### Supplementary Information

Supplementary Figures.

## Data Availability

The datasets analyzed during the current study were generated by the Therapeutically Applicable Research to Generate Effective Treatments (www.cancer.gov/ccg/research/genome-sequencing/target) (TARGET) initiative, phs000468, and are available in Genomic Data Commons repository for the Osteosarcoma TARGET Initiative (www.cancer.gov/ccg/research/genome-sequencing/target/studied-cancers/osteosarcoma). All additional data generated or analyzed during this study are included in this published article and/or will be available from the corresponding author on reasonable request.
