# Supplementary material for: IRF5 suppresses metastasis through the regulation of tumor-derived extracellular vesicles and pre-metastatic niche formation
Source: Sci Rep. 2024 Jul 5;14:15557. doi: 10.1038/s41598-024-66168-w (PMC11226449; doi:10.1038/s41598-024-66168-w)
Supplement: Supplementary file 1 — Supplementary Figures. [file 41598_2024_66168_MOESM1_ESM.docx]

**Supplemental Figure 1**

**Supplemental Figure 1:** Full Western Blot images from Figure 2. Western Blot images were obtained via Bio-Rad ChemiDoc Imager. Bands are imaged with HRP chemiluminescence detection and additional image of pre-stained ladder obtained with standard white light colorimetric imaging. In order to show full blots, chemiluminescent images were merged with colorimetric images. **(a)** Western blot for IRF5 in 4T1, 4T1-IRF5, K7M2 and K12 cells lines. **(b)** Same western blot as **(a)** for Actin expression. **(c)** Western blot for ALIX in K7M2, K12 EVs and 4T1 and 4T1-IRF5 EVs. **(d)** Western blot for CD63 in K7M2 and K12 EVs and 4T1 and 4T1-IRF5 EVs.

**Supplemental Figure 2**


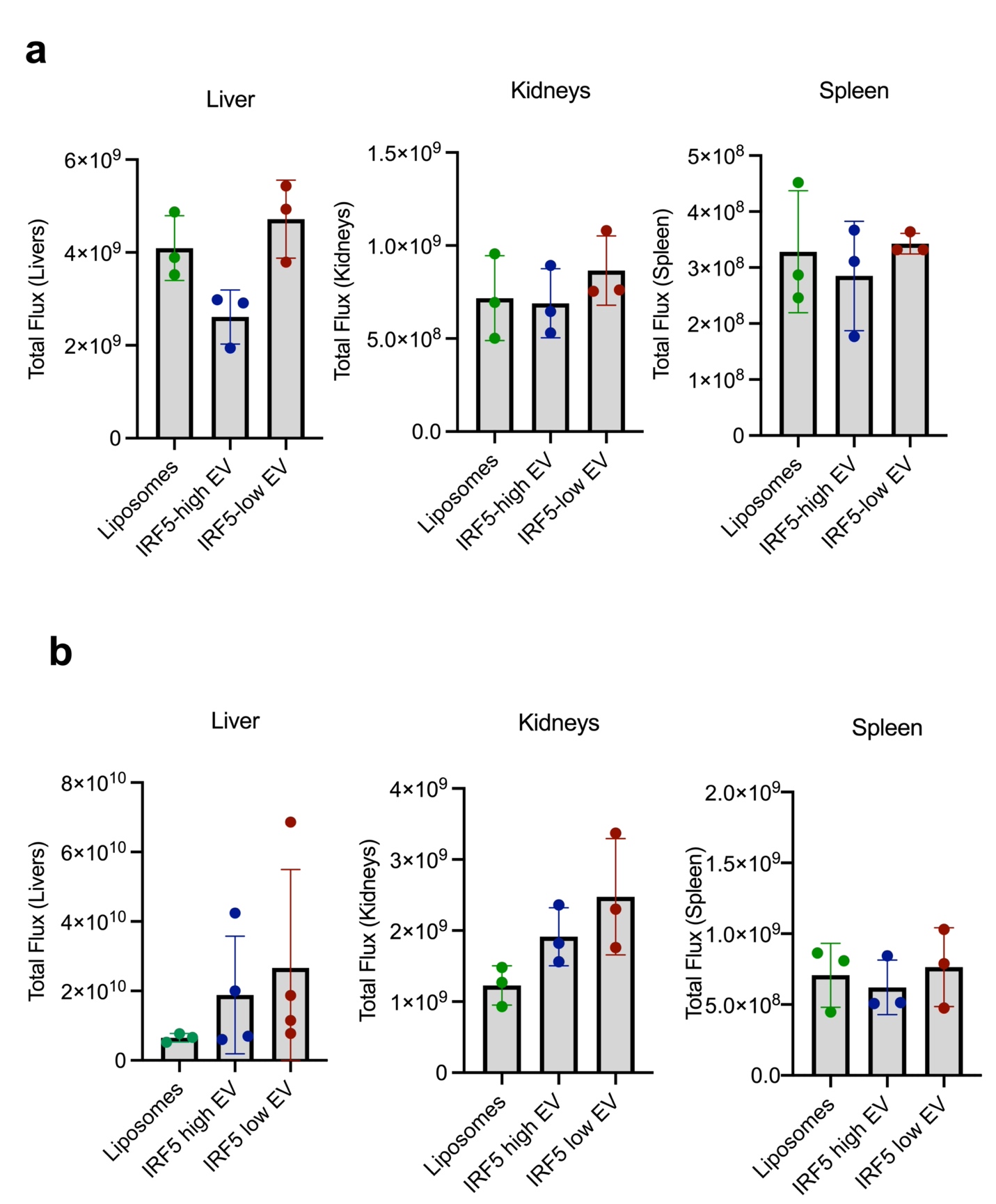


**Supplemental Figure 2:** Total flux in lung, kidneys and spleen of mice after tail vein injection of fluorescently-labeled t-dEVs or liposomes from OS **(a)** and BC **(b)**.

**Supplemental Figure 3**


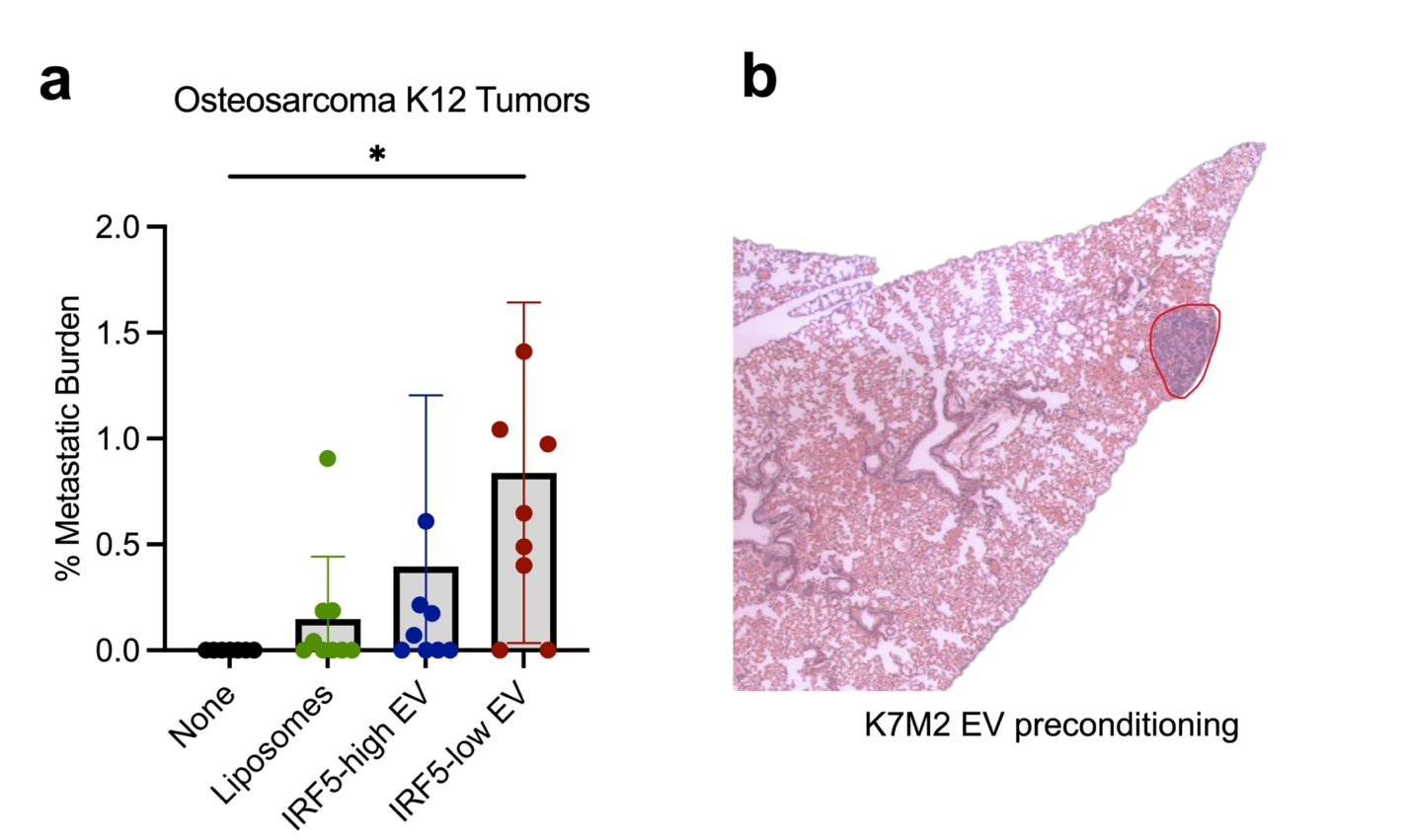


**Supplemental Figure 3:** **(a)** Quantitative differences in metastatic burden of mice pre-conditioned with nothing, liposomes, K12 EVs or K7M2 EVs prior to implantation of K12 tumors. **(b)** Representative H&E of metastatic burden in K7M2 EV pre-conditioned mouse.

**Supplemental Figure 4**


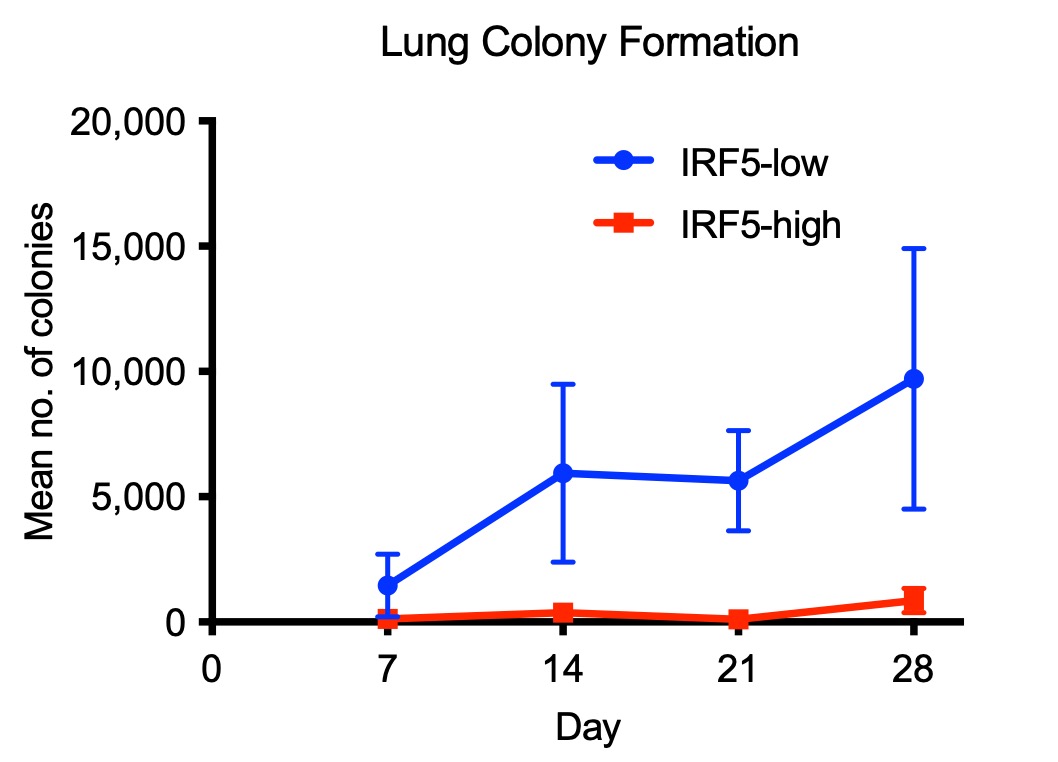


**Supplemental Figure 4:** Summarized data from lung colony formation assay from mice implanted with 4T1 IRF5-high or IRF5-low tumor cells.

**Supplemental Figure 5**

**
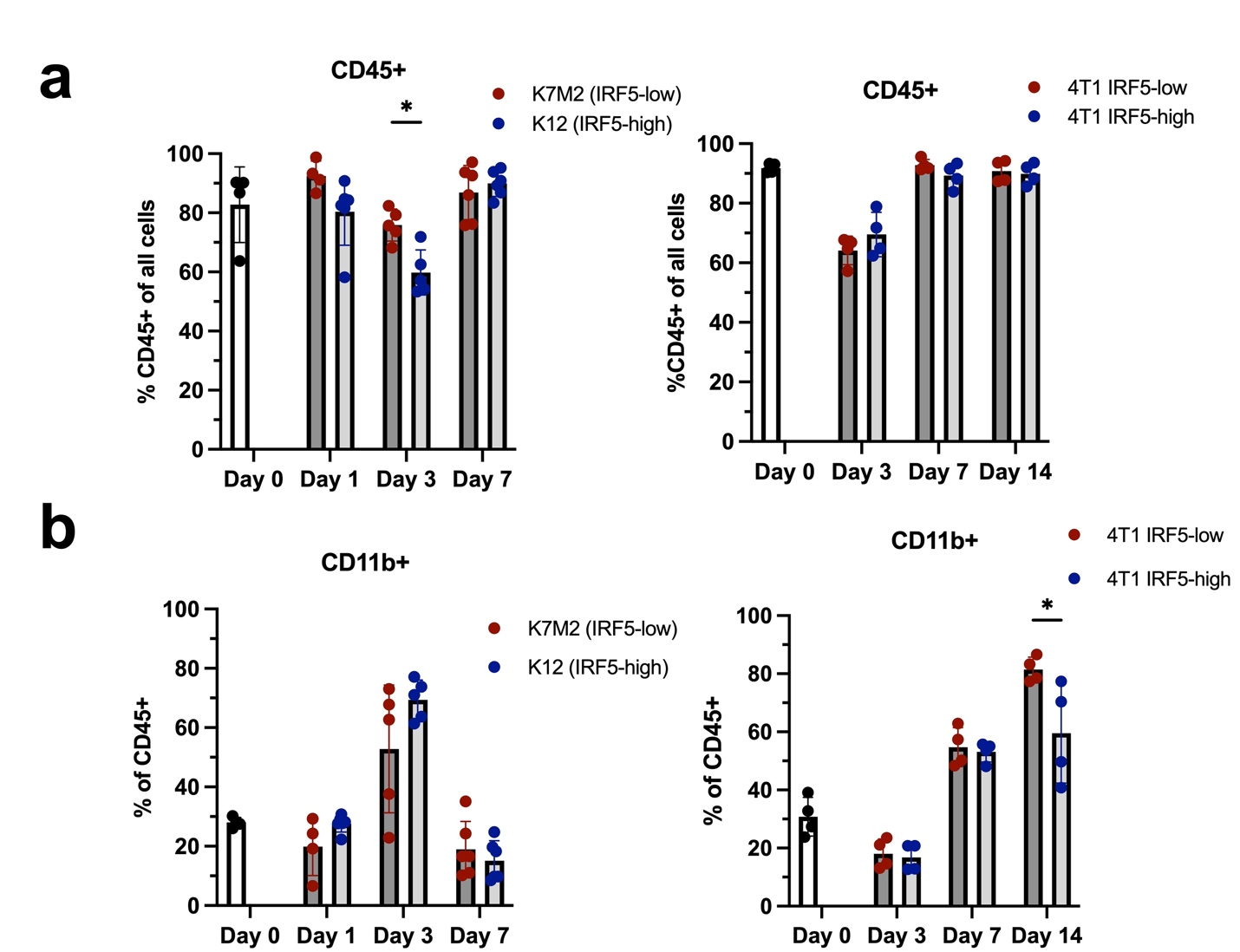
**

**Supplemental Figure 5:** (a) Quantitative differences between total lung CD45+ cells during early stages of PMN formation from mice implanted with OS or BC tumors. (b) Quantitative differences between total lung CD11b+ cells during early stages of PMN formation from mice implanted with OS or BC tumors.
